# Study on the Mechanism of Sancao Tiaowei Decoction in the Treatment of MNNG-Induced Precancerous Lesions of Gastric Carcinoma Through Hedgehog Signaling Pathway

**DOI:** 10.3389/fonc.2022.841553

**Published:** 2022-05-11

**Authors:** Yan Cai, Ying Cao, Shuang Cheng, Lijun Zou, Ting Yang, Yuxin Zhang, Qiyang Shou, Binhai Chen, Weijian Chen

**Affiliations:** ^1^The Second School of Clinical Medical, Zhejiang Chinese Medical University, Hangzhou, China; ^2^Oncology Department, Yuexi County Hospital, Anqing, China; ^3^School of Pharmaceutical Sciences, Zhejiang Chinese Medical University, Hangzhou, China; ^4^Oncology Department, The Second Affiliated Hospital of Zhejiang Chinese Medical University (Xinhua Hospital of Zhejiang Province), Hangzhou, China

**Keywords:** Sancao Tiaowei decoction, precancerous lesions of gastric carcinoma, hedgehog signaling pathway, multi-targeted, SMO (sliding mode observer), inflammation to cancer

## Abstract

Sancao Tiaowei Decoction (SCTWD), a traditional Chinese medicine created by Professor Chen Weijian, has been used in the prevention and treatment of precancerous lesions of gastric carcinoma (PLGC). However, its mechanism has not been made clear. This study aimed to evaluate the therapeutic effect of SCTWD on 1-methyl-3-nitro-1-nitrosoguanidine-induced PLGC in rats and the mechanism of this effect. We found that SCTWD effectively repaired gastric mucosal injury, reversed the process of PLGC, and inhibited the occurrence of gastric cancer to some extent. In the results of hematoxylin-eosin (HE) staining, the number and arrangement of mucosal glands and the number of mononuclear cells in the lamina propria were improved in varying degrees; the enzyme-linked immunosorbent assay (ELISA) showed that the PG I and PGR of the medication treatment group were significantly higher; a Reverse Transcription-Polymerase Chain Reaction (RT-PCR) test showed that SCTWD could significantly upregulate the expression levels of Shh, Ptch, and Gli-1 in the gastric tissue of rats. The immunohistochemical method showed that SCTWD could significantly upregulate the protein expressions of Shh, Gli-1, Smo, cyclin D1, CDKN2A/p16INK4a, and NF-κBP65 and could reduce the expression of Ptch at the same time. Through the preliminary analysis of 75 compounds screened by UPLC-Q-TOF-MS, the main components, such as organic acids, esters and anhydrides, flavonoids, phenols, tanshinones, and so on, have anti-inflammatory and anti-tumor pharmacological effects. The results of KEGG enrichment analysis showed that 5 signaling pathways related to this project were found, and 33 differential genes were presented to construct the interaction network. These results suggested that SCTWD had a good regulatory effect on PLGC and thus may have a multi-targeted effect; SCTWD can not only significantly improve the pathological changes of gastric mucosa in rats with PLGC but also exert a strong effect of the regulation of the hedgehog signaling pathway.

## Introduction

Precancerous lesions of gastric carcinoma (PLGC) are a kind of gastric mucosal histopathological changes that are prone to canceration, including gastric mucosal atrophy, intestinal metaplasia (IM), and dysplasia (Dys). Gastric mucosal atrophy and IM are collectively referred to as chronic atrophic gastritis (CAG) (1). According to the statistics from the literature, the probability of CAG, IM, and Dys developing into gastric cancer is monotonously increasing, with Dys being the highest, and approximately 1 in 19 people will develop gastric cancer within 20 years (2–4). With the growth of population in China, the number of patients with gastric precancerous lesions has increased significantly (5), and the trend of happening at a younger age is becoming more and more obvious. Currently, it’s generally believed that PLGC is irreversible and that the main purpose of the treatment is for the eradication of *Helicobacter pylori*, acid inhibition, the protection of the gastric mucosa, and symptomatic treatment (6), but the effect is not significant. Studies have shown that the hedgehog (Hh) signaling pathway in patients with PLGC is abnormally activated and has a specific expression in the process of the occurrence and development of PLGC (7).

Traditional Chinese medicine (TCM), which has a history spanning thousands of years, has attracted much attention in China and even the whole world. The research of TCM has become a research hotspot in the field of pharmacodynamics and pharmacokinetics. So far, TCM prescriptions have been widely used in the treatment of PLGC disease. For example, it has been reported that Weifuchun (WFC) tablets can regulate the expression of oncogenes and tumor suppressor genes by regulating nuclear factor kappa B (NF-κB), runt-related transcription factor 3/transforming growth factor-β/Smad (RUNX3/TGF-β/Smad), Hh, mitogen-activated protein kinase (MAPK), and Wingless and int-1 (Wnt) signaling pathways (8, 9). At the same time, erianin, the main active ingredient of *Dendrobium officinale Kimura et Migo*, inhibits PLGC through suppressing the Harvey rat sarcoma viral oncogene homolog/Phosphatidylinositol 3 Kinase/Protein Kinase B(HRAS/PI3K/AKT) signaling pathway (10). Similarly, the polysaccharides from *D. candidum* can reduce 8-OHdG levels and activate the NRF2 pathway and its related antioxidant enzymes HO-1 and NQO-1 to prevent PLGC and subsequent liver and kidney damage (11). A previous study reported that Xiaotan Hewei Decoction can also inhibit the activity of NF-κB and inhibit its transference from the cytoplasm to the nucleus (12). It is worth noting that, compared with the treatment plan and treatment mode of modern medicine, TCM can improve the clinical symptoms and gastric mucosal lesions of patients with PLGC to a certain extent. This new perspective of TCM research has made a contribution to modern medicine and has become a promising field of further research.

Sancao Tiaowei Decoction (SCTWD) is Professor Chen Weijian’s experienced prescription in the treatment of PLGC, which has achieved a good effect in clinical application. In the previous study of this project, the patients with PLGC of the Second Affiliated Hospital of Zhejiang Chinese Medical University who were treated by WC were selected to observe the clinical symptoms and histopathological changes before and after treatment with SCTWD.

The results show that SCTWD can not only improve the clinical symptoms of patients with PLGC but also improve the pathological state of the gastric mucosa, repair gastric mucosal injury, and reverse the process of PLGC to some degree in order to reduce the occurrence of gastric cancer. The purpose of this study is to determine the pharmacological effect of SCTWD on PLGC through the PLGC rat model, so as to clarify the therapeutic mechanism of SCTWD on PLGC through the Hh signaling pathway.

## Materials and Methods

### Experimental Animal

A total of 36 SD male rats, weighing (125 ± 5) g, were provided by Shanghai Shrek Experimental Animal Co., Ltd., of which animal production license number is: SCXK (Shanghai) 2017-0005; and whose animal use license number is: SYXK (Zhejiang) 2021-0012, raised in the Animal Experimental Research Center of Zhejiang Chinese Medical University, with relatively constant temperature (25°C), constant humidity (75%), and unlimited drinking water and feed.

### Ethical Review

This study has been examined and approved by the Experimental Ethics Committee of The Second Affiliated Hospital of Zhejiang Chinese Medical University and the Animal Experimental Ethics Committee of Zhejiang Chinese Medical University. The design meets the principles of safety and fairness. Experimental animals met the relevant national requirements for medical experimental animals.

### Drugs and Reagents

The traditional Chinese herbs of SCTWD were purchased in the pharmacy of The Second Affiliated Hospital of Zhejiang Chinese Medical University, and the quality was controlled according to the standard of the 2015 edition of *Chinese Pharmacopoeia*. SCTWD is composed of 9 herbs: *Pseudostellariae Radix*, stir-baked *Atractylodis Macrocephalae Rhizoma* in bran, *Poria*, *Agrimoniae Herba*, *Taraxaci Herba*, *Hedyotis Diffusa Willd*, *Salviae Miltiorrhizae Radix Etrhizoma*, *Curcumae Rhizoma*, and *Glycyrrhizae Radix Et Rhizoma*. The herbs were prepared by the laboratory of Zhejiang Chinese Medical University and soaked in water of which the volume was 8 times that of herbs for 1 hour. The traditional Chinese herbs were decocted twice with water whose volume was 20 times, mixed the two-time liquid of the medicine, concentrated into the liquid containing crude drug 2.8 g/ml by a rotary evaporator, which was sterilized and packed separately, then refrigerated for preparation.

1-Methyl-3-nitro-1-nitrosoguanidine (MNNG) was purchased from Hangzhou Northrend Biotechnology Co., Ltd, Hangzhou, Zhejiang, China. Ranitidine was purchased from Hangzhou Kuiteng Biotechnology Co., Ltd, Hangzhou, Zhejiang, China. Xylene (Item No.: R017750-500 ml) was purchased from Shanghai Yien Chemical Technology Co., Ltd, Shanghai, China. Hematoxylin stain Harris (Item No.: BL702A), eosin stain (alcohol solution) (Item No.: BL703A), Neutral Balsam (Item No.: BL704A), goat anti-mouse IgG- horseradish peroxidase (HRP) (Item No.: BL001A), goat anti-rabbit IgG-HRP (Item No.: BL003A) were purchased from Biosharp White Shark Biotechnology Co., Ltd, Hefei, Anhui, China. The rabbit kit (enhanced enzyme-labeled goat anti-rabbit IgG polymer) (Item No.: PV-9001), concentrated DAB kit (20 × 2 tubes) (Item No.: ZLI-9017), citrate buffer (Item No.: ZLI9065), and PBS phosphate buffer (Item No.: ZLI-9062) were purchased from Beijing Zhongshan Jinqiao Biotechnology Co., Ltd, Beijing, China. The RIPA lysis buffer (Item No.: P0013B), 4-20% SDS-PAGE pre-formed glue (Item No.: P0057A), Western primary antibody secondary antibody removal solution (Item No.: P0025-250ml), actin antibody (mouse monoclonal antibody) (Item No.: AA128), Gli-1 rabbit polyclonal antibody (Item No.: AF6990), Smo rabbit polyclonal antibody (Item No.: AF8010), PTCH1 rabbit polyclonal antibody (Item No.: AF7836), NF-kappa B p65 antibody (rabbit polyclonal antibody) (Item No.: AN365), cyclin D1 rabbit polyclonal antibody (Item No.: AF0126), CDKN2A/p16INK4a rabbit polyclonal antibody (Item No.: AF6471), and HRP-labeled goat anti-rabbit IgG (Hendl) (Item No.: A0208) were purchased from Shanghai Biyuntian Biotechnology Co., Ltd, Shanghai, China. PageRuler Prestained Protein Ladder, 10–180 kDa (Item No.: 26617) was purchased from Thermo Fisher Scientific Company, Waltham, Massachusetts, the United States; ammonium hydroxide (Batch No.: 20010328) was purchased from Hangzhou Changzheng Chemical Plant, Hangzhou, Zhejiang, China; sodium chloride (Batch No.: 060304) was purchased from Shanghai No.4 Reagent & H.v.chemical Co., Ltd, Shanghai, China ; 3% H2O2 solution (Batch No.: 200508292) was purchased from Shanghai Broad Peroxide Company, Shanghai, China; and methanol (Batch No.: 200608010) was purchased from Shanghai Zhenxing No.1 Chemical Plant Co., Ltd, Shanghai, China. Anhydrous formic acid (Batch No.: 040518) was purchased from Shanghai Lingfeng Chemical Reagent Co., Ltd, Shanghai, China; concentrated hydrochloric acid (Batch No.: 20050603) and ethanol (Batch No.: 20050521) were purchased from Hangzhou Chemical Reagent Co., Ltd, Hangzhou, Zhejiang, China; paraformaldehyde (Batch No.: 20051208) was purchased from Tianjin Institute of Chemical Testing Agents Quzhou Juhua Co., Ltd, Tianjin, China; the neutral formaldehyde buffer solution (Batch No.: 20060101) was purchased from Quzhou Juhua Co., Ltd, Quzhou, Zhejiang, China. The rabbit anti-patched antibody (Item No.: bs-1614R) is purchased from Beijing Boosen Biotechnology Co., Ltd, Beijing, China; the Shh antibody (item No.: 20697-1-AP) was purchased from Proteintech Group Co., Ltd, Chicago, Illinois State, the United States. The rat serum TNF-α enzyme-linked immunosorbent assay (ELISA) kit (Item No.: BEP30635), rat serum pepsinogen I (PG I) ELISA kit (Item No.: BEP30821), rat serum pepsinogen II (PG II) ELISA kit (Item No.: BEP30896), and rat serum gastrin (GAS) ELISA kit (Item No.: BEP30675) were purchased from Shanghai Langton Biotechnology Co., Ltd, Shanghai, China. The rat IL-6 kit (Item No.: AB234570) was purchased from Abcam Company Co., Ltd, Cambridge, the United Kingdom. The rat IL-8 ELISA kit (Item No.: SEKR-0071) was purchased from Shanghai Solebo Technology Co., Ltd, Shanghai, China. While IL-8 Antibody-Internal (Item No.: DF6698) and IL-6 Antibody-Internal (Item No.: DF6087) were purchased from Affinity Biosciences Co., Ltd, Cincinnati, State of Ohio, the United States. The RNA extraction kit (Item No.: AHF1991D), RT reverse transcription kit (Item No.: RR036A), SYBR Green fluorescent quantitative PCR dye (Item No.: AK9301) were purchased from TaKaRa Bio Co., Ltd, Tokyo, Japan.

## Method

### Drug Analysis by UPLC-Q-TOF-MS

*Pseudostellariae Radix* 15 g, stir-baked *Atractylodis Macrocephalae Rhizoma* in bran 15 g, *Poria* 15 g, *Agrimoniae Herba* 15 g, *Taraxaci Herba* 15 g, *Hedyotis Diffusa Willd* 15 g, *Salviae Miltiorrhizae Radix Etrhizoma* 15 g, *Curcumae Rhizoma* 9 g, and *Glycyrrhizae Radix Et Rhizoma* 9 g were immersed for 30 min in 10 times volume of pure water, decocted and extracted twice for 1 hour each time, and the filtrate was concentrated to obtain the extract with the mass concentration of 1.23 g/ml. Approximately 1.0 ml of the extract was taken, which was put into the 100 ml measuring bottle, and deionized water was added for 30-minute ultrasonic. Deionized water was added to fix the volume until the concentrated solution is completely dissolved, it was centrifuged for 20 min with 14,000 r/min, and the supernatant was filtered through a 0.2 μm microporous membrane. All samples were analyzed by UPLC-Q-TOF/MS high-resolution four-stage flight tandem liquid chromatography–mass spectrometry (American model Waters SYNAPTG2-Si) for positive and negative ion mode analysis.

*Chromatographic conditions*: Eclipse Plus C18 column (50 mm × 2.1 mm, 1.8 μm) was selected as the chromatographic column. The separated mobile phase consists of solvent A (water, 0.1% formic acid) and solvent B (acetonitrile, 0.1% formic acid). The gradient elution conditions are as follows: gradient elution is used (elution procedure: 0–2 min, 5% solvent B; 2–32 min, 5%–100% solvent B; 32–33 min, 100% solvent B; 33.5 min, 5% solvent B), and the later running time is 5 min. The injection volume is 2 μl, the temperature of the column incubator is 35°C, and the temperature of the automatic injector is 10°C.

*Mass spectrometry conditions*: The ESI positive ion mode and negative ion mode were used to collect data. The range of scanning (m/z) was 50–1,200 Da, the capillary voltage of positive ion mode was 3,000 V as well as the negative ion mode was 2,500 V, the source offset voltage was 80 V, with the taper hole voltage 40 V and the atomizer pressure 6.0 Bar, the source temperature was 100°C, the desolvent temperature was 400°C, and the desolvent gas flow rate was 800 L/h.

*Experimental instrument*: UPLC-Q-TOF-MS (specification: SYNAPT G2mursi; model: Waters SYNAPT G2-Si) was purchased from Waters Corporation, Milford, Massachusetts, United States.

### Grouping, Modeling, and Intervention

Thirty-six 3-day-old male suckling rats were selected and divided into cages by the random number table method after routine feeding to 3-week-old weaning. A total of 8 suckling rats were divided into the blank group and free diet. The other 28 suckling rats were used as model rats, and the model group was fed with nitrosoguanidine carcinogen combined with ranitidine feed. Approximately 420 ml of pure water mixed with 80 ml of alcohol and 5 g of nitrosoguanidine formed the mother liquid of 10 mg/ml, and the drinking water containing 100 μg/ml of medicine was prepared according to 1:100 once a week, combined with 0.03% ranitidine feed. The model group was fed, following the rule “full for two days and fasting for one day”. The model was made continuously by the above-mentioned method for 10 weeks. In the model group, 2 rats were randomly selected for anesthesia, and the stomach was taken for pathological examination every 2 weeks. After the establishment of the model, the model rats were randomly divided into two groups: the model group (n = 9) and treatment of SCTWD group (n = 9). The pathology was reviewed and checked by senior pathologists to determine gastric mucosal atrophy, and IM and IN were seen at the same time; then, the PLGC model was judged to be successful.

Referring to 60 kg, the average body mass of adults, according to the dose conversion between the clinical drug dose and the animal dose, the dose given SCTWD daily with raw medicine was 22.4 g/kg (8 ml/kg). The model group was given physiological saline, according to the body weight of rats. The intragastric treatment of 0.1 ml/(kg·d) was performed. Drug intervention lasted for 16 weeks.

### Record the Changes of Weight of Rats

The rats were weighed and recorded every other day before the establishment of the model and during the experiment, and the changes of weight were observed.

### Collection and Treatment of Specimens

After giving the last medicine, the rats were treated with fasting for solids and liquids for 12 h and then anaesthetized with 3% pentobarbital sodium; 10 ml of blood was collected from the celiac vein and stored at 4°C. The 5 ml separated serum was packed in 3 parts, stored in the refrigerator at -80°C. The gastric tissue was cut along the great curvature of the stomach with the gastric contents removed, and spread out for general observation. The gastric tissue at the boundary of the lesser curved sinus was taken for general observation, with one part fixed with 4% paraformaldehyde, as well as the other three parts were preserved at -8°C, and the alcohol was dehydrated step by step, transparented by xylene, embedded by paraffin, and made routinely into paraffin sections to be used.

### Index Detection

#### Observation of Histopathological Changes by HE Staining

After the gastric body was embedded in paraffin, the gastric body was sliced with slicer and stained with HE, and the histopathology of gastric mucosa of rats in each group was observed under light microscope.

#### Detection of the Expression of Ptch, Shh, and Gli-1 mRNA in Gastric Tissue by RT-PCR

After grinding, RNA was extracted with Trizol reagent and trichloromethane, precipitated with isopropanol, washed with 75% ethanol, dried and dissolved in a proper volume (30~50 μl) of diethyl carbonate containing coke (DEPC), and stored at -80°C. Total RNA was extracted directly for PCR amplification. According to the instructions of the M-MuLV first-strand cDNA synthesis kit, the reverse transcription was complement deoxyribonucleic acid (cDNA). After the reverse transcription was completed and using this as a template, THUNDERBIRD SYBR qPCR Mix and specific primers were used to synthesize the corresponding PCR products. The specific operation steps refer to the instructions of the kit. Heat the product for 10 min at 70°C to end the reaction, and put it on ice for follow-up experiments or cryopreservation. The RT-PCR reaction conditions were pre-denatured at 94°C, 5 min; at 94°C, 50 s; at 58°C, 50 s; at 72°C, 1 min. Approximately 8 min was extended at 72°C after 35 cycles. The Ptch reaction procedure was pre-denatured at 94°C, 5 min: at 94°C, 50 s; at 54°C, 50 s; at 72°C, 1 min. Approximately 8 min was extended at 72°C after 35 cycles.

Experimental instrument:1300 Series A2 Class II, Type A2 Biological Safety Cabinets, High Speed Refrigerated Centrifuge, Ultra-Low Freezers and Nanodrop Trace Nucleic Acid Tester were purchased from American Thermo Fisher Scientific company, Waltham, Massachusetts, the United States. Homogenizer was purchased from Bertin Technologies, Aix-en-Provence, France. Automatic Fluorescence Quantitative PCR Instrument was purchased from American ABI company, Waltham, Massachusetts, the United States. was purchased from Bio-Rad Laboratories, Hercules, California, the United States. SIM-F140AY65-PC Ice Maker was purchased from Panasonic company, Osaka, Japan. Pipette was purchased from Eppendorf company, Hamburg, Germany. UVITEC Ultraviolet Gel Imaging Systemwas purchased from British UVITEC Company, Cambridge, the United Kingdom.

#### Detection of Serum PG I and PG II by Enzyme-Linked Immunosorbent Assay, and Calculation of the Ratio of PG I/PG II (PGR)

The levels of PG I and PG II in the blood of rats were detected by double-antibody-sandwich ELISA, and the ratio of PGR was calculated. The specific operation was carried out according to the instructions of the kit.

Experimental instrument: Multiskan FC Microplate Photometer was purchased from American Thermo Fisher Scientific company, Waltham, Massachusetts, the United States. Plate washer was purchased from Bio-Rad Laboratories, Hercules, California, the United States. Liquid Transfer Machine was purchased from Eppendorf Company, Hamburg, Germany.

#### Detection of the Expression of IL-6, IL-8, Smo, Ptch, Shh, Gli-1, Cyclin D1, CDKN2A/p16INK4a, and NF-κBp65 Protein in Gastric Mucosa by Immunohistochemical Method

After paraffin section, using xylene for dewaxing to water, gradient rehydration and high pressure antigen repair, immunohistochemical staining was performed according to the instructions of the kit. PBST buffer staining was used as negative control. Five images of each tissue about mucous layer were randomly collected under a ×200 microscope, and the images were analyzed by Image Pro Plus 6.0 software.

Experimental instrument: Nikon Eclipse 80i Microscope nd DS-H1 500 Pixel CCD were purchased from Nikon Corporation, Tokyo, Japan. Carl Zeiss lmaging Systems was purchased from Carl Zeiss Company, Oberkochen, Germany. Microwave oven was purchased from Guangdong Galanz Enterprises Co., Ltd, Foshan, Guangdong, China. DRP-9052 type electric thermostat was purchased from Shanghai Senxin Experimental Instrument Co., Ltd, Shanghai, China. BG-270 waterproof electric thermostat was purchased from Medical Equipment Factory of Shanghai Medical Instruments Co., Ltd, Shanghai, China.

### Analysis of Rat Gastric Tissue by Reference Transcriptome Sequencing

In this project, the difference multiple FC ≥ 2 or FC ≤ 0.5 (the same as the absolute value of log_2_ FC ≥ 1) was used as the change threshold, and *P* < 0.05 was used as the criterion for screening differential genes. In the set comparison group, the results of differentially expressed genes were obtained, and differential genes were screened to construct interaction networks.

### Statistical Analysis

All the data were analyzed by SPSS 26.0 (SPSS Inc., Chicago, IL, the United States), and expressed by mean ± standard error (SEM). The measurement data were evaluated by ANOVA analysis of variance, and pairwise comparison was conducted by the Dunnet test. The values of the statistical results were retained in the last 2 decimal places. For data analysis and processing, each group is allowed to eliminate no more than 2 abnormal data (beyond the range of mean ± 2SD and abnormal experimental conditions). The difference (*P* < 0.05) was statistically significant. The UPLC-Q-TOF-MS method was analyzed by Waters Mass Lynx 4.1 and MSE Data Viewer 1.2 software.

## Result

### Analysis of Chemical Constituents in Compound Prescription by UPLC-Q-TOF-MS

**Figure 1:**
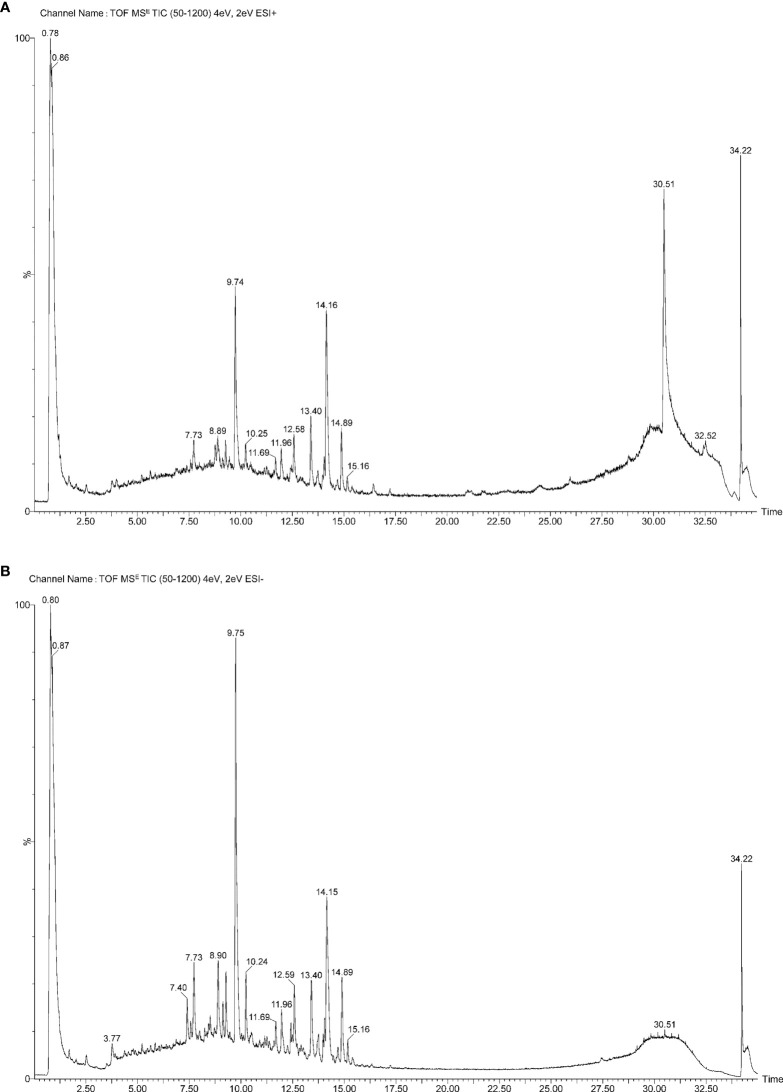
Analysis of chemical constituents in compound prescription by UPLC-Q-TOF-MS. **(A)** The primary total ion flow diagram of SCTWD under positive ion mode. **(B)** The primary total ion flow diagram of SCTWD under negative ion mode.

The water extract of SCTWD was detected by the UPLC-Q-TOF-MS method, and the results showed that there were 75 components in the water extract; the results are shown in **Table 1**, including organic acids, esters and anhydrides, flavonoids, phenols, tanshinones and other major components. Modern pharmacological studies have shown that their main components have anti-inflammatory and anti-tumor effects. The primary total ion flow diagram of SCTWD under positive ion mode is shown in **Figure 1A**, and that of negative ion mode is shown in **Figure 1B**.

**Table 1:** Characteristics of some chemical components in SCTWD by UPLC-Q/TOF MS analysis.

Name	Formula	Molecular weight	RT(min)
Dimethyl lithospermate	C29H26O12	566.1424	1.66
Monomethyl lithospermate	C28H24O12	552.1268	0.99
Genkwanin	C16H12O5	284.0685	7.66
Licurazide	C26H30O13	550.1686	5.83
Serine	C3H7NO3	105.0426	1.08
Salviolone	C18H20O2	268.1463	17.23
3-(4-Hydroxy-3-methoxyphenyl)propionic acid	C10H12O4	196.0736	4.37
Formononetin	C16H12O4	268.0736	8.06
Furfuryl alcohol	C5H6O2	98.03678	0.83
Ethyl caffeate	C11H12O4	208.0736	6.01
Trans-p-Coumaric Acid	C9H8O3	164.0473	1.21
Salvianolic acid A	C26H22O10	494.1213	8.02
Cryptoacetalide	C18H22O3	286.1569	9.23
Arucadiol	C19H22O3	298.1569	12.29
Miltionone I	C19H20O4	312.1362	8.46
Artemitin	C20H20O8	388.1158	7.69
4,5-O-Dicaffeoylquinic acid	C25H24O12	516.1268	0.81
13-Hydroxygermacrone	C15H22O2	234.162	15.74
Caffeic acid	C9H8O4	180.0423	9.75
3β,16α-Dihydroxylanosta-7,9(11),24-trien-21-oic acid	C30H46O4	470.3396	14.89
4,5-O-Dicaffeoylquinic acid	C25H24O12	516.1268	10.88
Scopoletin	C10H8O4	192.0423	6.31
7-Methoxycoumarin	C10H8O3	176.0473	6.72
Isoetin	C15H10O7	302.0427	9.56
Isoetin-7-O-β-D-glucopyranosyl-2’-O-β-D-xyloypyranoside	C26H28O16	596.1377	5.96
Rufescidride	C18H8O7	336.027	8.67
Phenylalanine	C9H11NO2	165.079	5.82
Atractylenolide I	C15H18O2	230.1307	15.88
Miltionone I	C19H20O4	312.1362	7.8
Miltionone II	C19H20O4	312.1362	12.5
Tanshinone IIB	C19H20O3	296.1412	17.23
Salvianolic acid A	C26H22O10	494.1213	10.92
Salvianolic acid C	C26H20O10	492.1057	10.25
Salvianolic acid G	C18H12O7	340.0583	8.91
4-Hydroxybenzoic acid	C7H6O3	138.0317	4.33
Anti-coumaric acid	C9H8O3	164.0473	1.2
Liquiritin	C21H22O9	418.1264	9.47
Glabrolide	C30H44O4	468.324	13.41
Liquiritigenin	C15H12O4	256.0736	6.04
Glycyrrhizic acid	C42H62O16	822.4038	15.17
L-glutamic acid	C5H9NO4	147.0532	0.87
Quercetin	C15H10O7	302.0427	8.48
Isoquercitrin	C21H20O12	464.0955	7.85
Quercetin-7-O-β-D-glucopyranosyl(1→6)-β-D-glucopyranoside	C27H30O17	626.1483	5.98
Baicalin	C21H18O11	446.0849	8.61
Curcolone	C15H18O3	246.1256	13.14
L(+)-Arginine	C6H14N4O2	174.1117	0.8
Leucine	C6H13NO2	131.0946	1.33
Rutin	C27H30O16	610.1534	7.72
Chlorogenic acid	C16H18O9	354.0951	5.23
Formononetin	C16H12O4	268.0736	10.45
Luteolin-7-O-β-D-glucoside	C21H20O11	448.1006	8.71
Zederone	C15H18O3	246.1256	12.88
DL-Proline	C5H9NO2	115.0633	0.83
Taraxacin	C15H14O3	242.0943	8.78
Hydroxytanshinone IIA	C19H18O4	310.1205	15.62
Apigenin	C15H10O5	270.0528	8.61
Daphnetin	C9H6O4	178.0266	5.51
7-Hydroxycoumarine	C9H6O3	162.0317	6.17
Kaempferol	C15H10O6	286.0477	8.65
Bisdemethoxycurcumin	C19H16O4	308.1049	16.54
Heterophyllin A	C37H57N7O8	727.4269	13.86
Linderazulene	C15H14O	210.1045	16.44
Adenosine	C10H13N5O4	267.0968	1.21
Valine	C5H11NO2	117.079	0.94
Neoliquiritin	C21H22O9	418.1264	9.72
Isocurcumenol	C15H22O2	234.162	20.18
Neolicuroside	C26H30O13	550.1686	7.73
Isoliquiritin	C21H22O9	418.1264	7.73
Isoliquiritigenin	C15H12O4	256.0736	8.35
Isoglabrolide	C30H44O4	468.324	12.59
Genkwanin-4’-O-β -D-lutinoside	C28H32O14	592.1792	10.51
Protocatechualdehyde	C7H6O3	138.0317	8.9
Lithospermic acid	C27H22O12	538.1111	10.25
Lithospermic acid B	C36H30O16	718.1534	9.75

### Observation and Study on the Clinical Curative Effect

**Figure 2:**
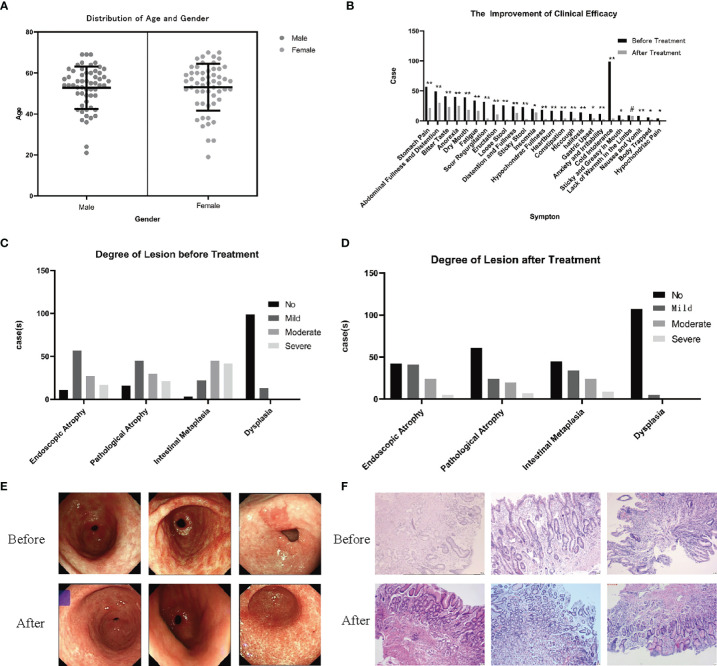
Observation and study on the clinical curative effect in the previous study. **(A)** Gender and age distribution of 112 patients with PLGC (*P* > 0.05). **(B)** After 6 months of treatment with SCTWD, the clinical symptoms of the patients improved (^*^P < 0.01, ^**^P < 0.05, ^#^*P* > 0.05). **(C)** Histopathological distribution of patients before treatment. **(D)** Histopathological distribution of patients after treatment. The histopathological distribution of patients before and after treatment data were analyzed by ANOVA (*P*<0.01). **(E)** Gastroscopic pictures of 3 patients before and after treatment were showed, and all the observation sites were antrum. **(F)** Histopathological sections of 3 patients before and after treatment were taken from the gastric antrum. Scale bar: 20 µm.

In the previous study of this project, 112 patients with PLGC were selected from The Second Affiliated Hospital of Zhejiang Chinese Medical University, which is the outpatient clinic of Professor Chen Weijian, from January 2019 to June 2020, as shown in **Figure 2**, of which 49.11% (55 cases) were women and 50.89% (57 cases) were men. The average age of the patients was 55 (13) years old. The sex and age distribution were shown in **Figure 2A**, including 96 cases of pathological atrophy, 109 cases of IM and 13 cases of Dys. There was no significant difference in age between men and women (*P* > 0.05). After 6 months of treatment with SCTWD, the clinical symptoms and histopathological changes of the patients were significantly improved. The results of clinical symptom improvement are shown in **Figure 2B**, and the histopathological results before and after treatment are shown in **Figures 2C, D**. Stomach pain, abdominal distension and fullness, bitter taste, anorexia, dry mouth, fatigue, sour regurgitation, eructation, loose stool, distention and fullness, sticky stool, hypochondriac fullness, heartburn, constipation, hiccough, halitosis, anxiety and irritability, as well as nausea, and vomiting were significantly different between before and after treatment (*P* < 0.01); there was significant difference in symptom before and after treatment, such as insomnia, gastric upset, sticky and greasy in mouth, body trapped, and hypochondriac pain (*P* < 0.05); the lack of warmth in the limbs was no significant difference between before and after treatment (*P* > 0.05). The total effective rate of symptom improvement is 91.96%; the total effective rate of atrophy is 62.89%; the total effective rate of intestinal metaplasia is 74.31%; and the total effective rate of dysplasia is 61.54%. The gastroscope and pathology of the patients before and after treatment are shown in **Figures 2E, F**.

### Changes of Weight and Gastric Mucosa in Rats

#### Changes of Weight in Rats

**Figure 3:**
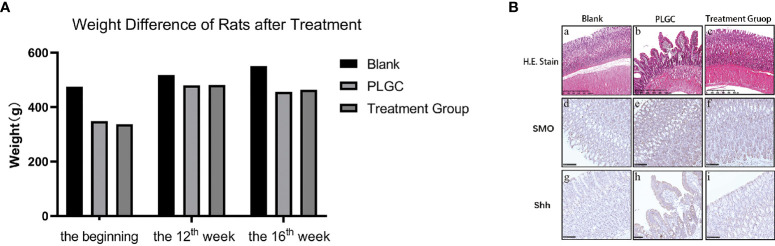
Changes of weight and gastric mucosa in rats. **(A)** Weight difference of rats at the beginning of treatment and the 12th week and 16th week of treatment. **(B)** The pathological changes of gastric tissue sections stained with HE were observed. Scale bar: 500 µm.

The difference of weight of rats weight difference of rats at the beginning of treatment and the 12th week and 16th week of treatment were calculated, respectively, and the difference of weight is shown in **Figure 3A**.

#### Histological Observation of Gastric Mucosa

The glandular stomach of rats in the blank group had no obvious change; the mucous membrane was covered with more mucus and the mucosa was ruddy, while in the model group, the glandular stomach had some changes, the mucosal surface was yellow, and the color of exposed mucosa was slightly whiter than that in the blank group.

#### Histopathological Observation of HE Staining

The results are shown in **Figure 3B**. In the blank group, the mucosal structure was normal, the glands were arranged tightly and neatly, with the structure complete, and the shape and size were basically the same. There was no inflammatory cell infiltration, and the submucosa and muscle layer were continuous, which was the normal pathological manifestation of gastric tissue (**Figure 3B: a, d, g**). In the model group, gastric mucosa atrophied to varying degrees, and the mucosal layer became thinner, with the muscularis mucosa thickened and the lamina propria glands decreased, as well as the arrangement was sparse and irregular. Some glands were dilated, with the nucleus stained deeply, as well as the shapes and sizes were different, and the glandular duct was incomplete. An obvious infiltration of lymphocytes and plasma cells was able to be seen, accompanied by IM and IN (**Figure 3B: b, e, h**). The results of microscopic observation showed that the PLGC rat model was constructed successfully. Compared with the model group, the pathological results from the medication treatment group were improved in varying degrees, with the number of mucosal glands increased and arranged neatly and the number of mononuclear cells in lamina propria decreased (**Figure 3B: c, f, i**).

### Detection of the Expressions of Shh, Ptch and Gli-1 mRNA in Gastric Tissue by RT-PCR

**Figure 4:**
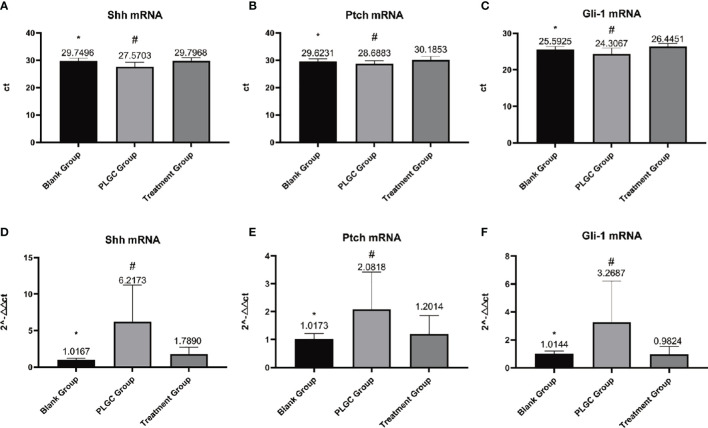
Detection of the expression of Shh, Ptch, and Gli-1 mRNA in gastric tissue by RT-PCR. **(A)** Expression of Shh mRNA in each group. **(B)** Expression of Ptch mRNA in each group. **(C)** Expression of Gli-1 mRNA in each group. **(D)** Relative quantitative expression of Shh mRNA in each group. **(E)** Relative quantitative expression of Ptch mRNA in each group. **(F)** Relative quantitative expression of Gli-1 mRNA in each group. ^*^P < 0.05 vs. blank; ^#^*P* < 0.01 vs. PLGC.

The results are shown in **Figure 4**. Compared with the blank group, the expressions of Shh, Ptch, and Gli-1 mRNA in the gastric tissue of the model group decreased, but compared with the model group, the expression of mRNA in the medication treatment group increased. Comparing the expressions of Shh mRNA, there was a significant difference between the model group and the treatment group (*P* < 0.01), and there was no significant difference between the treatment group, the model group and blank group (*P* > 0.05, **Figures 4A, D**); Comparing the expressions of Ptch mRNA, there was a significant difference between the model group and the treatment group, model group, and blank group (*P* < 0.01), and there was no significant difference between the treatment group and the blank group (*P* > 0.05, *P* = 0.097, **Figures 4B, E**); Comparing Gli-1 mRNA expression, there was a significant difference between the model group and treatment group, blank group, and model group (*P* < 0.01), and there was no significant difference between the blank group and the treatment group (*P* > 0.05, *P*=0.996, **Figures 4C, F**).

### Comparison of Serum PG I, PG II, and PGR in Rats of Each Group

**Figure 5:**
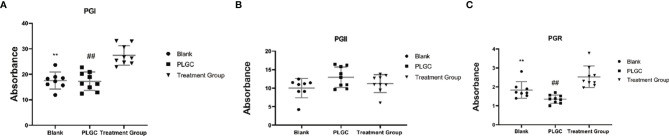
Comparison of serum PG I, PG II, and PGR in the rats of each group. **(A)** The level of serum PG I in each group. **(B)** The level of serum PG II in each group. **(C)** The level of serum PGR in each group. **P < 0.01 vs. Blank; ^##^*P* < 0.01 vs. PLGC.

The results are shown in **Figure 5**. Compared with the model group, there was no significant difference in serum PG I, PG II, and PGR between the blank group and the model group. The serum levels of PG I (**Figure 5A**) and PGR in the medication treatment group were significantly higher than those in the model group (*P* < 0.01, **Figure 5C**), as well as there was no significant difference in the serum levels of PG II in the groups (**Figure 5B**).

### Comparison of Expression Levels of Shh, Ptch, Gli-1, Cyclin D1, CDKN2A/p16INK4a, NF-κBp65, Smo, IL-6, and IL-8 protein in Gastric Tissue of Rats in Different Groups

**Figure 6:**
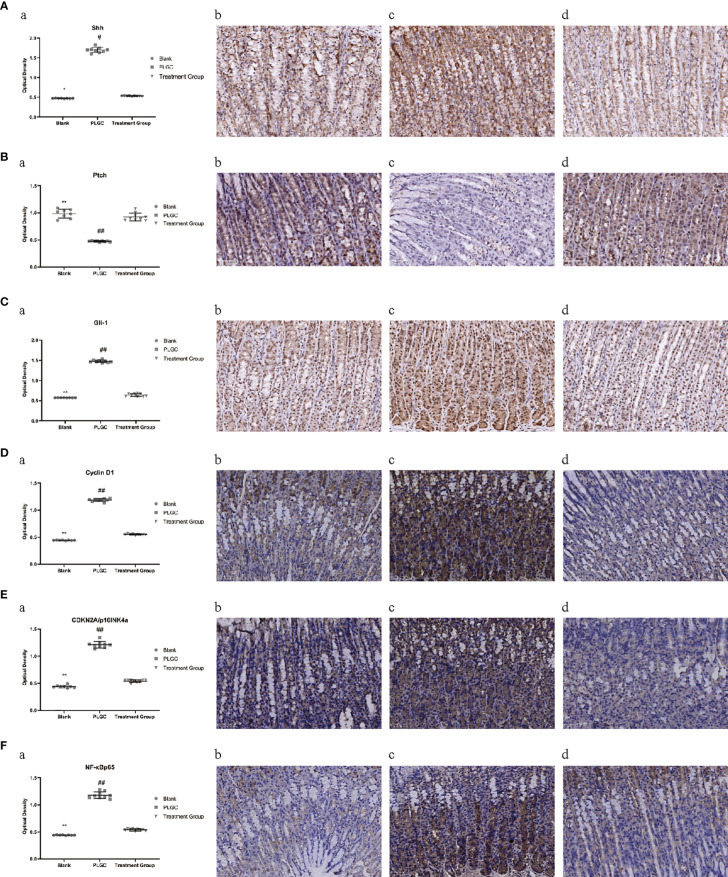
Comparison of expression levels of Shh, Ptch, Gli-1, Cyclin D1, CDKN2A/p16INK4a, and NF-κBp65 protein in the gastric tissue of rats in different groups. **(A)** The expression level of Shh protein in the gastric tissue of rats in each group was detected by immunohistochemistry. **(B)** The expression level of Ptch protein in the gastric tissue of rats in each group was detected by immunohistochemistry. **(C)** The expression level of Gli-1 protein in the gastric tissue of rats in each group was detected by immunohistochemistry. **(D)** The expression level of cyclin D1 protein in the gastric tissue of rats in each group was detected by immunohistochemistry. **(E)** The expression level of CDKN2A/p16INK4a protein in the gastric tissue of rats in each group was detected by immunohistochemistry. **(F)** The expression level of NF-κBp65 protein in the gastric tissue of rats in each group was detected by immunohistochemistry. ^*^P < 0.05 vs. blank; ^**^P < 0.01 vs. blank; ^#^*P* < 0.05 vs. PLGC; ^##^*P* < 0.01 vs. PLGC. Scale bar: 50µm.

The results are shown in **Figures 6A–F**. Compared with the blank group, the expressions of Gli-1, CyclinD1, CDKN2A/p16INK4a and NF-κBp65 protein in the gastric tissue of the model group were significantly increased, but the gene expressions of the medication treatment group were lower than that of the model group (*P* < 0.01). Meanwhile, compared with the blank group, the expressions of Shh protein in the gastric tissue of the model group were increased, but the gene expressions of the medication treatment group were lower than that of the model group (*P* < 0.05). On the contrary, the expression of Ptch was significantly decreased in the model group and upregulated in the medication treatment group (*P* < 0.05).

#### Comparison of Expression Levels of Smo, IL-6, and IL-8 in Gastric Tissue of Rats in Different Groups

**Figure 7:**
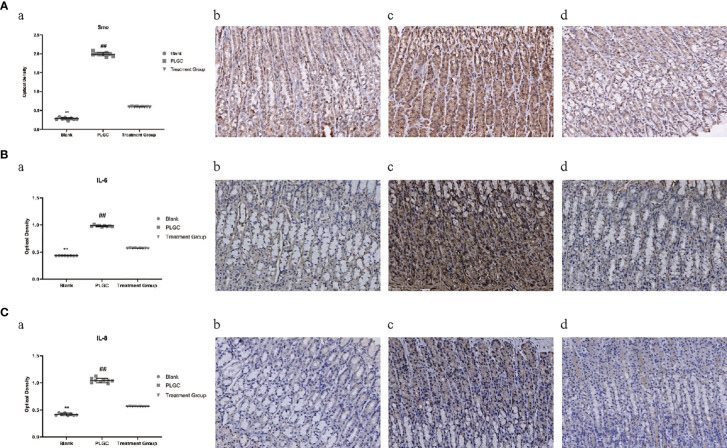
Comparison of expression levels of Smo, IL-6, and IL-8 protein in the gastric tissue of rats in different groups. **(A)** The expression of Smo in the gastric tissue of rats was detected by immunohistochemistry. **(B)** The expression of IL-6 in the gastric tissue of rats was detected by immunohistochemistry. **(C)** The expression of IL-8 in the gastric tissue of rats was detected by immunohistochemistry. **P < 0.01 vs. Blank; ^##^*P* < 0.01 vs. PLGC. Scale bar: 50 µm.

The positive expression of Smo was mainly located on the cell membrane and in the cytoplasm. Microscopically, positive cells were expressed in the mucous layer, mucosal muscular layer, muscular layer, and serous layer of gastric tissue. Compared with the blank group, the expression of Smo, interleukin-6 (IL-6) and interleukin-8 (IL-8) protein in the gastric tissue of the model group increased, but compared with the model group, the gene expression of the medication treatment group decreased significantly(*P* < 0.01, **Figures 7A–C**).

### Differential Gene Expression

#### Differential Gene Expression Level Analysis and Cluster Analysis

**Figure 8:**
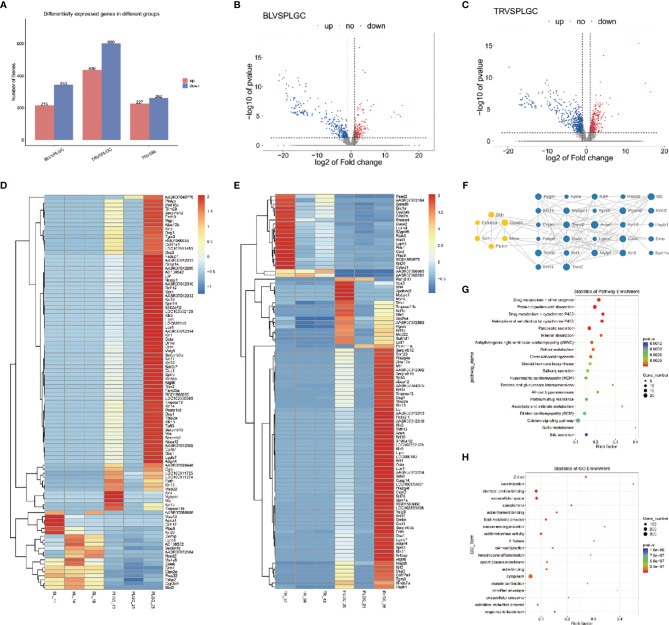
Differential gene expression. **(A)** Statistics of upregulated and downregulated differentially expressed genes in each group. **(B)** Distribution of upregulated and downregulated differentially expressed genes in the blank group and model group. **(C)** Distribution of upregulated and downregulated differentially expressed genes between the treatment group and the model group. **(D)** Heat map was used to show the gene expression in the blank group and model group. **(E)** Heat map was used to show the gene expression in the treatment group and model group. **(F)** Interaction network constructed by 33 genes. **(G)** Make KEGG enrichment analysis scatter diagram according to the significance of enrichment. **(H)** Make GO enrichment analysis scatter diagram according to the significance of enrichment.

In this project, we counted the number of differential genes in all the comparison groups with the difference multiple FC ≥ 2 or FC ≤ 0.5 (the same as the absolute value of log_2_ FC ≥ 1) as the threshold and *P* < 0.05 was used as the criterion for screening differential genes to count the number of differential genes in all comparison groups. Comparing the model group to the blank group, the results showed that 343 genes were downregulated and 215 genes were upregulated. A total of 600 genes were downregulated and 436 genes were upregulated upon comparing the medication treatment group with the model group, which was shown in a bar chart (**Figure 8A**). The overall distribution of differentially expressed genes was shown by a volcanic diagram (**Figures 8B, C**). Cluster analysis is a method of multivariate classification and statistical analysis. In order to better reflect the clustering expression pattern, a heat map is used to judge the regulation pattern of genes under different experimental conditions and to directly show the expression of genes in different treatments (**Figures 8D, E**). Using the string database and Cytoscape software, the first 30 significantly different genes were screened out to build an interaction network with 5 tested genes in this study (two genes have no interaction with other genes), as shown in **Figure 8F**.

#### GO Function and KEGG Pathway Annotation of Differentially Expressed Genes

According to the Kyoto Encyclopedia of Genome and Genome (KEGG) classification map, five signaling pathways related to this project are shown in the **Figure 8G**. Gene Ontology (GO) is an international standardized classification system of gene function, which can comprehensively describe the attributes of genes and gene products in organisms and significantly enrich GO entries. The results of GO enrichment analysis are shown in the form of a scatter diagram (bubble chart) (**Figure 8H**). The top three places of significant enrichment are identical protein binding, the extracellular space, and cytoplasm.

## Discussion

In the literature of TCM, there is no clear discussion on PLGC, which can be classified into the categories of “ruffian fullness,” “epigastric pain,” “stomach ruffian,” and “gastric discomfort” according to its clinical manifestations. Professor Chen Weijian believes that the pathogenic factors of PLGC are complex and diverse, such as the invasion of exogenous pathogen, the internal injury of diet, emotional disorder, and the weakness of spleen and stomach, which can cause the ascension and descension of spleen and stomach and the dysfunction of the qi of the middle energizer. The disease is located in the stomach and is closely related to the spleen and liver. The disease is mostly based on the deficiency in origin and excess in superficiality, intermingled deficiency and excess: the deficiency of spleen and stomach, spleen qi deficiency and stomach yin deficiency, and the internal resistance of exogenous pathogen, such as blood stasis, qi stagnation, and dampness-heat. Its basic pathogenesis is the weakness of spleen and stomach, the stagnation of qi, blood, and phlegm, which obstructs stomach collaterals, leading to the loss of the nourishment of the stomach body and the onset of the disease. The pathological manifestations were abnormal metabolism of gastric mucosal cells, the atrophy of the gastric gland, IM, and even low-grade intraepithelial neoplasia, which eventually developed to gastric malignant tumor.

Some TCM scholars believe that the mechanism of PLGC is the process of “inflammation to cancer” (13). The “invasion of exogenous pathogen” caused by *H. pylori* infection and “improper diet” are the main causes of “inflammatory-cancer transformation” (14). Xiaolin Tong put forward the “State-Target” Strategy and believed that chronic gastritis was based on spleen deficiency and marked by phlegm dampness, blood stasis, and qi stagnation (15). Xijie Zhu believed that PLGC is the result of anoxia and the proliferation of gastric mucosa due to the repeated invasion of disease pathogens (16). The key to treatment is to dispel disease pathogens, taking “disease pathogens” as treatment targets, and multi-level differentiation and treatment. Professor Correa proposed the basic pathway of gastric cancer: chronic non-atrophic gastritis→ CAG→ CAG with IM→ CAG with Dys/atypical hyperplasia/intraepithelial neoplasia→ gastric cancer (17). This approach is consistent with the theoretical model of “inflammation to cancer.”

SCTWD is Professor Chen Weijian’s experienced prescription in the treatment of PLGC, which mainly aims at the syndrome of spleen deficiency and qi stagnation along with phlegm and blood stasis. This prescription is composed of *Pseudostellariae Radix* , stir-baked *Atractylodis Macrocephalae Rhizoma* in bran, *Poria*, *Agrimoniae Herba*, *Taraxaci Herba*, *Hedyotis Diffusa Willd*, *Salviae Miltiorrhizae Radix Etrhizoma*, *Curcumae Rhizoma*, and *Glycyrrhizae Radix Et Rhizoma*. *Pseudostellariae Radix* and stir-baked *Atractylodis Macrocephalae Rhizoma* in bran, as monarch medicine, *Pseudostellariae Radix* returns to the heart, lung and spleen meridians, which can tonify spleen and lung, tonify qi, and refresh and enrich the saliva, and is the representative of moistening tonification; stir-baked *Atractylodis Macrocephalae Rhizoma* in bran is able to tonify spleen, invigorate spleen, and permeate dampness, which not only helps *Pseudostellariae Radix* tonify spleen and stomach qi but also helps invigorate spleen dryness and dampness with its bitter temperature. The two herbs are used to support acquired foundation, and spleen is well functioned to invigorate water and dampness, as well as regulating the qi of the spleen and stomach can consolidate the foundation, and the five internal organs can be recuperated. *Taraxaci Herba* has the function of clearing heat and detoxification, scattering tumescence, and loosening the knot. *A New Edition of Chinese Materia Medica* shows that *Taraxaci Herba* can purge fire without damaging the soil (which, in the TCM, refers to spleen), so it can be taken for a long time without hindrance. *Hedyotis Diffusa Willd* has a taste of bitter cold, which has the effect of clearing heat, detoxification, and dampness. The taste of *Agrimoniae Herba* Ledeb is bitter and astringent, and it has the function of astringent hemostasis, detoxification, and tonifying deficiency. *Poria* enters the heart, lung, spleen, and kidney meridians, which are sweet and can tonify, lighten, and permeate turbid toxin. All three herbs mentioned above are ministerial drugs, which are combined with *Pseudostellariae Radix* and stir-baked *Atractylodis Macrocephalae Rhizoma*e in bran to tonify without stagnation, to clear heat, and to detoxify to remove dampness. *Experience Gained in Treating External Diseases* shows that “Tumor is formed by blood stasis, turbid qi and phlegm stagnation of five internal organs. *Curcumae Rhizoma* has the function of breaking blood, removing blood stasis and relievig pain, and *Salviae Miltiorrhizae Radix Etrhizoma* has the function of promoting blood circulation, removing blood stasis, and relieving menstruation and pain, which are adjuvant drugs. *Glycyrrhizae Radix Et Rhizoma*, as an adjuvant and messenger drug, is to tonify spleen and middle qi, help *Pseudostellariae Radix* and stir-baked *Atractylodis Macrocephalae Rhizoma* in bran nourishing middle warmer and tonifying qi, and even become more adjustable and compatible with all kinds of drugs. The combination of various medicines can play the role of eliminating and tonifying which shows the effects of strengthening the spleen and stomach, regulating qi, resolving phlegm, removing blood stasis and promoting tissue regeneration.” Modern pharmacological studies have shown that *Agrimoniae Herba*, *Taraxaci Herba*, *Hedyotis Diffusa Willd*, *Salviae Miltiorrhizae Radix Etrhizoma* and *Curcumae Rhizoma* all have the effects of treating gastrointestinal diseases and anti-cancer. *Taraxaci Herba* extract has a strong ability to activate delayed anaphylaxis of tumor cells and can inhibit the invasion and migration of gastric cancer cells (18). Agrimoniin extracted from *Agrimoniae Herba* has anti-tumor activity to promote tumor cell cycle arrest, to induce apoptosis, and to enhance the immune function of the body (19). *Hedyotis Diffusa Willd* contains a variety of anti-cancer chemical components, and its mechanisms include the inhibition of tumor cell proliferation, the induction of apoptosis, the enhancement of immunity, anti-tumor angiogenesis, and so on (20). Curcumol can inhibit BGC-823, a kind of human gastric cancer cell, and induce its apoptosis (21). Tanshinone II A can inhibit SGC7901, which is a kind of human gastric cancer cell, reduces the expression of cyclooxygenase-2 (COX-2) and inhibit tumor invasion and metastasis (22). SCTWD has achieved a good effect in clinical application, which can not only improve the clinical symptoms of patients with PLGC but also improve the pathological state of gastric mucosa, repair gastric mucosal injury, and reverse the process of PLGC.

TCM has been used for thousands of years, and its safety has been proven to be high. The TCM contained in this prescription is commonly used in clinical practice. No death or other side effects have been found after consulting the data and reports. Under the condition of clinical safety, we converted the dose of clinical medication with reference to the average adult weight of 60 kg into the experimental concentration of rats and there were no side effects or death that happened throughout the experiment. The three doses were 11.2 g/kg (4 ml/kg), 22.4 g/kg (8 ml/kg), and 44.8 g/kg (16 ml/kg) with raw medicine. All the three concentrations included the range of safety. Based on the results of ELISA experiment, we chose the middle-dose group for other experiments because its effect is the best out of the three groups.

**Figure 9:**
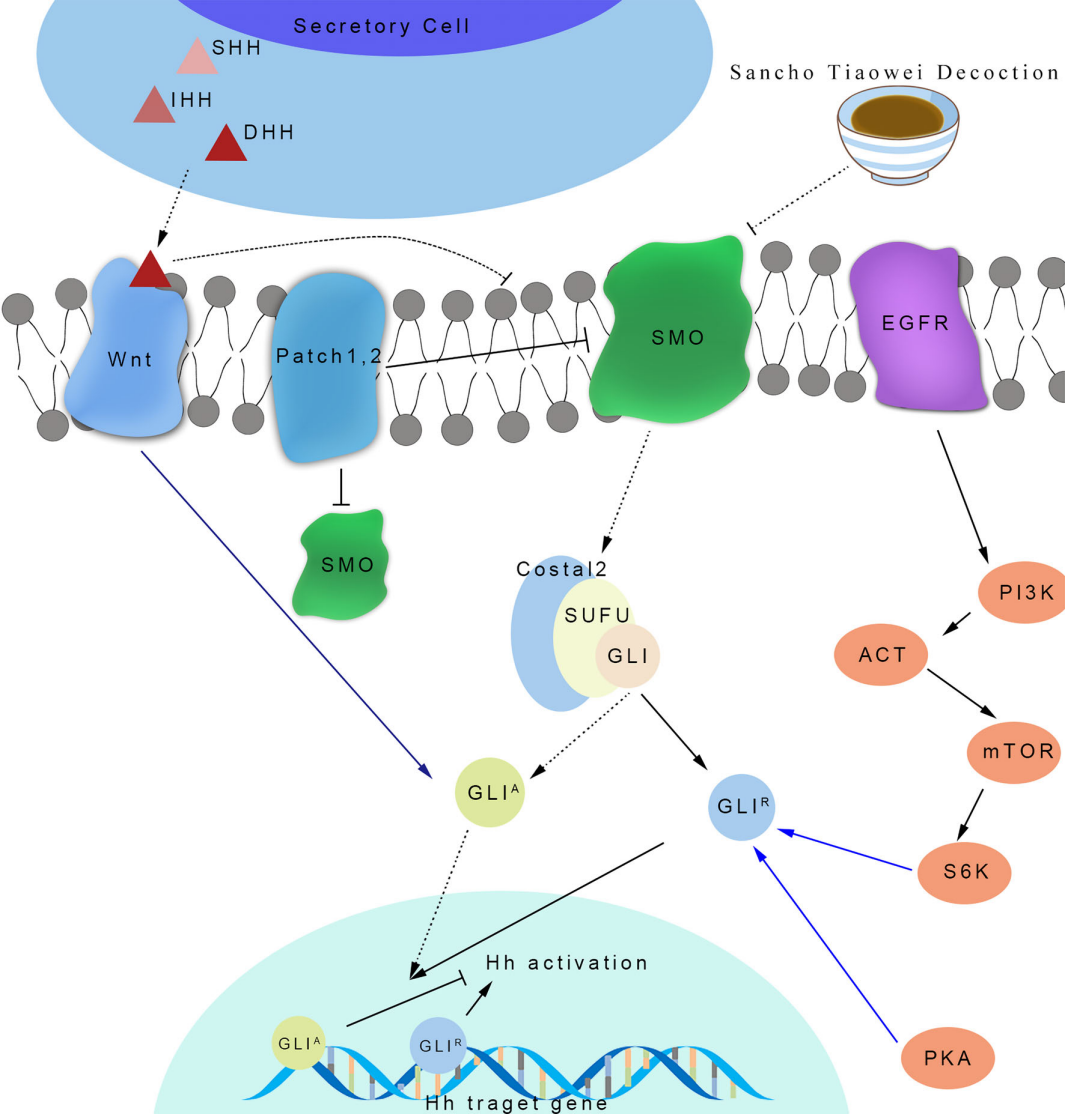
The mechanism of SCTWD in the treatment of PLGC through hedgehog signaling pathway.

In the occurrence of PLGC, the existence of the Hh signaling pathway (23) is very important (**Figure 9**). After the activation of the Hh signaling pathway, it inhibits the expression of the gene Ptch, in order to form a negative feedback regulation loop, which maintains the stability of the signaling pathway and regulates the normal growth and proliferation of cells. When the Hh signaling pathway is abnormally activated and out of control, it is continuously activated and the nuclear target genes are transcribed, which leads to uncontrolled cell growth and proliferation, leading to PLGC. The Hh signaling pathway includes ligand protein Hh, Ptch protein, Smo protein, and cytoplasmic protein complexes. The ligand protein Hh includes Sonic hedgehog (Shh), Indian hedgehog (Ihh), and desert hedgehog (Dhh). The cytoplasmic protein complex involved in Hh signaling transduction is mainly composed of Fu, SuFu, and zinc-finger transcription factor Gli (24). Gli (including Gli-1, Gli-2, and Gli-3) is the key protein that activates or inhibits the transcription of target genes in the Hh signaling pathway. In the Hh signaling pathway, Hh, Smo, and Fu play a positive regulatory role, while Ptch and SuFu play a negative regulatory role. Gli is the key molecule to function and can directly regulate the transcription of downstream target genes after entering the nucleus. The Hh signaling pathway can control gastric cell proliferation, differentiation, and gastric acid secretion by regulating the proliferation and differentiation of human stem cells, especially the Smo protein, which plays an important role in Shh signaling (25). The Shh signaling pathway is mainly composed of the Shh ligand, Smo protein complex, transmembrane protein receptor Ptch, and transcription factor Gli protein. When atrophy and dysplasia gradually appear in gastric mucosa, the Shh signaling pathway is activated and combined with Ptch, which relieves the inhibition of Ptch. The expressions of Shh, Smo, Gli-1, and Cyclin D1 mRNA in gastric mucosa increase in varying degrees. Smo activates downstream transcription factor Gli and initiates the transcription of many target genes (7). Knocking out the Smo gene, as a key signaling transduction molecule of the Hh signaling pathway, will block the Hh signaling pathway in rat embryos (26). Pepsinogen is a precursor of the digestive enzymes secreted by the digestive glands of gastric mucosa. According to its immunogenicity, pepsinogen can be divided into two subgroups: PG I and PG II. It has been proven that the expression of PG I decreases during the occurrence of PLGC, and PGR, the ratio of PG I to PG II, decreases, so PG I, PG II, and PGR can reflect gastric mucosal atrophy (27).

This study found that the Hh signaling pathway is activated in the occurrence and development of PLGC. After the intervention of SCTWD, the expression levels of key factors that are Shh, Smo, Gli-1, and so on in the signaling pathway are downregulated, while the expression of Ptch is upregulated, indicating that SCTWD can regulate the activation of the Hh signaling pathway by regulating the protein expression levels of multiple target molecules in the Hh signaling pathway, improve the pathological changes of gastric mucosa, and inhibit the progression of PLGC in PLGC rats.

Currently, it’s generally believed that PLGC is an irreversible and refractory disease because of the lack of specific and effective drugs in clinical practice. The current treatment methods can only delay the process, which means that there is no effective standard treatment strategy. TCM is more effective than western medicine in improving clinical symptoms, repairing mucosal injury, and reversing PLGC gastric gland atrophy and IM. In recent years, more and more scholars have found that the Hh signaling pathway plays an important role in cell proliferation, cell differentiation, and organ development during embryonic development, and its abnormal activation is related to a variety of human cancers. The multi-target effect of TCM is the main development direction of TCM in the treatment of PLGC to prevent the occurrence of gastric cancer. It has become a new trend to clarify the pathogenesis of PLGC and the effect mechanism of prescription and medicine.

## Conclusion

This study points out that spleen and stomach weakness and qi–blood–phlegm–dampness-blocking stomach collaterals are the basic pathogenesis of PLGC. Taking “invigorating the spleen and stomach, regulating qi, resolving phlegm, removing blood stasis and regenerating” as the treatment principle, the concept of strengthening the body resistance to eliminate pathogenic factors through its treatment, while regulating the expressions of proteins related to the Hh signaling pathway, is the main basis of the prescription of SCTWD for the differentiation and treatment of PLGC. In conclusion, SCTWD has a significant regulatory effect on MNNG-induced PLGC and may have a multi-targeted effect. SCTWD can not only significantly improve the pathological changes of gastric mucosa in rats with PLGC but also exert a strong effect of the regulation of the Hh signaling pathway.

## Data Availability Statement

The datasets presented in this study can be found in online repositories. The names of the repository/repositories and accession number(s) can be found below: https://figshare.com/, 10.6084/m9.figshare.17311556.

## Ethics Statement

The studies involving human participants were reviewed and approved by the Experimental Ethics Committee of The Second Affiliated Hospital of Zhejiang Chinese Medical University. The patients/participants provided their written informed consent to participate in this study. The animal study was reviewed and approved by Animal Committee of the Animal Experimental Center of Zhejiang Chinese Medical University.

## Author Contributions

YaC, YiC, SC, LZ, and QS conceived and designed the study. YaC, YiC, YZ, and TY performed the experiments. YaC and YiC analyzed the data. WC and BC contributed reagents and materials. YaC and YiC wrote the manuscript. All authors contributed to the article and approved the submitted version.

## Funding

This study was funded by the Traditional Chinese Medicine Modernization Special Project of Zhejiang Province (Project No.: 2022ZX007).

## Conflict of Interest

The authors declare that the research was conducted in the absence of any commercial or financial relationships that could be construed as a potential conflict of interest.

## Publisher’s Note

All claims expressed in this article are solely those of the authors and do not necessarily represent those of their affiliated organizations, or those of the publisher, the editors and the reviewers. Any product that may be evaluated in this article, or claim that may be made by its manufacturer, is not guaranteed or endorsed by the publisher.
